# Head to Toe Psychiatry: The Lady Who Limped in front of a Psychiatrist

**DOI:** 10.1155/2023/5642798

**Published:** 2023-03-27

**Authors:** Emmanuel Stip, Hind Mohammed Ahmed, Syed Fahad Javaid, Leena Amiri

**Affiliations:** ^1^Department of Psychiatry and Behavioral Sciences, College of Medicine and Health Sciences, United Arab Emirates University, Al Ain, UAE; ^2^Department of Psychiatry, University of Montréal, Canada; ^3^Behavioral Sciences Institute, Al Ain Hospital, Al Ain, UAE

## Abstract

Physical examination is a core component of any assessment done by a physician. Despite that, a physical examination is not always a top priority in many patients with psychiatric illnesses. We present the case of a woman with a prior diagnosis of a delusional disorder with overinvested religious beliefs. The patient had been stable on treatment for many years and only recently presented with a physical complaint, and manifestation assumed to be due to the nature of her psychiatric illness and, hence, overlooked by many physicians before being examined by her last psychiatrist. This resulted in a significant mobility problem for the patient. The patient showed partial insight, linking her pain to a “message from God.” Despite the delusional context, the psychiatrist was allowed to examine her feet and discovered significant neglect and poor foot hygiene. This case emphasizes the importance of conducting thorough physical examinations in psychiatric settings. Moreover, it presents an example of situations preventing psychiatric patients from being examined despite displaying obvious physical signs.

## 1. Introduction

Psychiatry is perceived to be a specialty that is mainly concerned with disorders of the mind. Unfortunately, psychiatrists do not always see physical examination as a critical part of the patient assessment and do not routinely examine their patients [1–3]. Although it is part of the standard psychiatric evaluation, the mental state examination nearly always trumps a physical examination when evaluating someone with a mental health disorder. Moreover, many psychiatrists do not always have the confidence to perform a thorough physical examination and believe that this task is better performed by a nonpsychiatric physician [1].

In practice, the majority of psychiatrists will resolve to examine their patients if there is a raised suspicion of a neurological condition causing the present psychiatric manifestations or when the exclusion of side effects of psychotropic medications needs to be considered as it can be the window to a more serious outcome like neuroleptic malignant syndrome.

In some cases, the patient might not cooperate with the examination in an acute setting due to their behavioral presentation. Such cases will require following the visual cues and observing the patient looking for any abnormality or even admitting the patient for further assessment when the patient permits.

In this report, the patient expressed and displayed discomfort in her lower extremities. However, it was never explored until her new psychiatrist noticed her gait abnormality, which prompted him to investigate and discover an underlying physical issue.

## 2. Case Presentation

We report the case of a 72-year-old woman who had been under psychiatric care for more than a decade. She lived alone and would visit her children once every two months. Nevertheless, she would socialize with her neighbors from time to time. Financially, she had a limited budget, yet it was sufficient for her basic needs. Her appetite was fair and so was her daily food intake. Her apparent hygiene levels were appropriate. She came across as a deeply religious person and was observed by the neighbors praying loudly and talking to herself.

Because of her underlying delusions of persecution and prejudice, she was seen by a psychiatrist who treated her symptoms with a small dose of antipsychotic (olanzapine 5 mg a day). Her symptoms were well-controlled, with no history of exacerbations, relapses, or hospitalization. There was no viscosity, no hypergraphia, and no circumstantiality. There were no noted side effects or changes in her biological markers throughout her treatment, as confirmed by her routine laboratory investigations.

The psychiatrist enjoyed a good rapport with the patient. This led to her opening up and talking more about her religious beliefs. At the end of each visit, she used to say to that psychiatrist, “You are treating me, but it is God who heals.” There were no reported symptoms of anxiety, phobias, or obsessions. Her mood was overall euthymic, and her sleep was sufficient and satisfactory. The patient did not state any issues or concerns and attended her clinic appointment primarily for a medication refill.

Her religious beliefs became more evident in the weeks leading to her psychiatric review. She was noted asking for forgiveness for her sins and believed that the pain in her leg was a punishment from God, and she fully accepted this act of atonement. When she told the psychiatrist about her beliefs, the option of increasing the dose of her medication was discussed.

The consultation in question was with a new psychiatrist and took place during the winter months. Due to the cold weather, she was covered up well and wore boots. On her way to the consultation office, she was noticed to be limping and reported that it had been the case for several months, attributing it to the pain inflicted by God for redemption from her sins. She had already talked to her general physician and the nurses about the pain.

In the psychiatrist's office, she further elaborated on her delusional religious beliefs. The lady strongly connected her lower limb pain and her beliefs of being punished by God. The psychiatrist then started discussing her gait abnormality for the first time and confronted her with the possibility that the pain and limping could be explained by reasons other than what she believed. She was not receptive to other explanations for her limp. The psychiatrist then explained to her the need for a physical examination to exclude having side effects from her medication like akathisia or other extrapyramidal symptoms.

**Figure 1:**
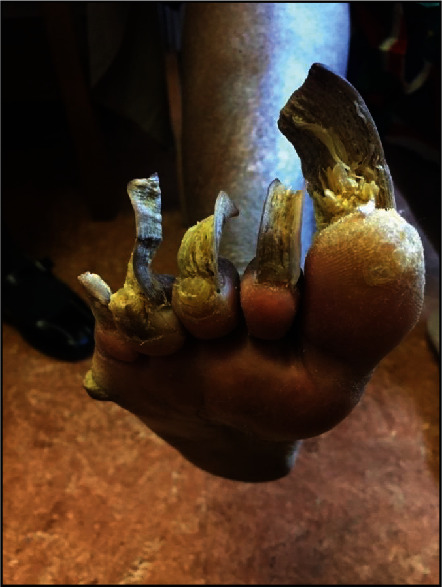
Plantar view of the patient's right foot. Note the overgrown toenails and a visible callus laterally.

**Figure 2:**
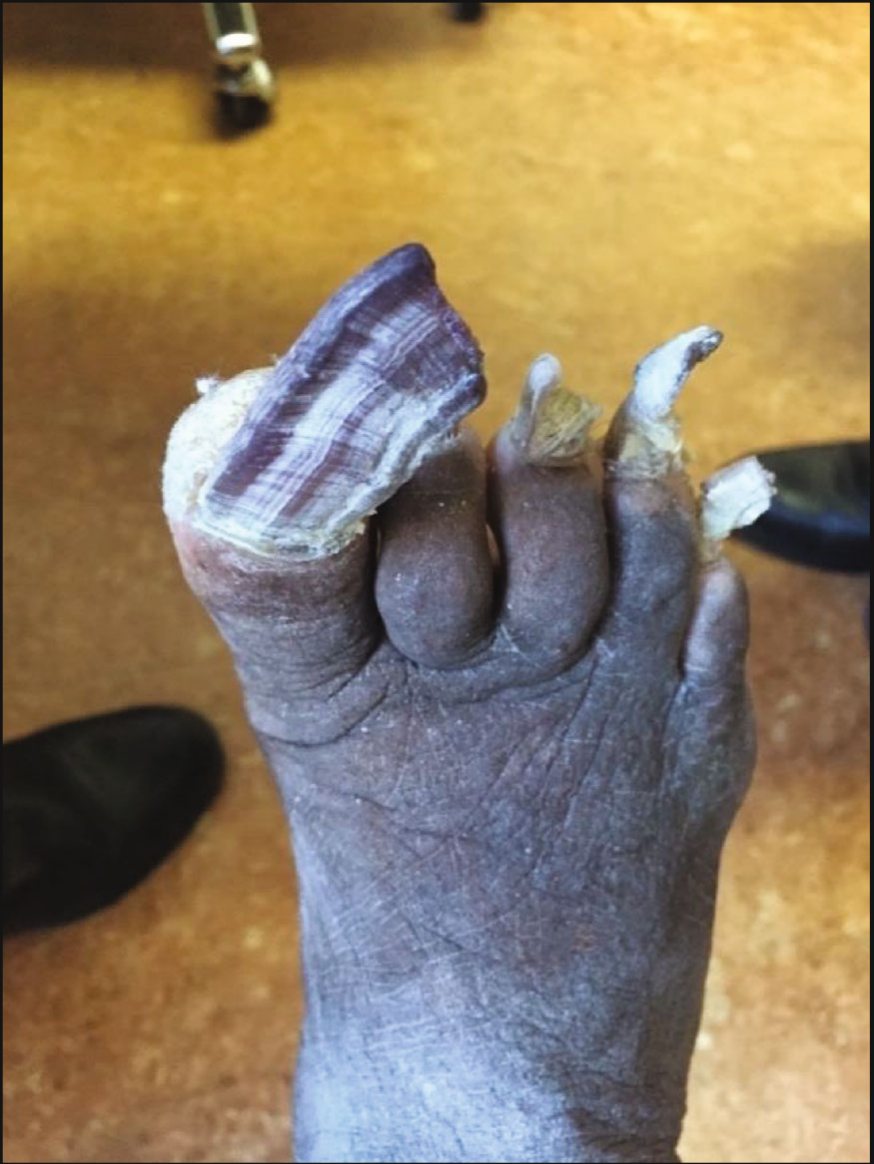
Dorsal view of the patient's right foot. Note the overgrown toenails and dry skin.

Before allowing him to take off her boots, she asked the psychiatrist if he was a “real doctor.” After reassurance, he gently took off her socks and found her feet in a state of total neglect, as shown in Figures 1 and 2.

Following the inspection, the psychiatrist explained to the patient that the pain and gait abnormality were most likely because of the state of her toenails that led to inadequate space inside her boots. This finding made the psychiatrist question her lifestyle, personal care, social life, and support, especially in relation to her children. He called the nurses to support the patient; she was transferred to the Emergency Department for urgent care.

The patient was thoroughly examined in the Emergency Department, and the doctor made the same observations. However, there were no urgent foot care services available in the Emergency Department. The mental health team made several phone calls before finally reaching a podiatrist who agreed to see the patient the next day. The foot specialist had his work cut out with our patient, who had not cut her toenails in over a year and needed urgent treatment. It was noted that one of the patient's toenails had grown to the size of 7 centimeters. The woman's thick, yellow fingernails took around 50 minutes to cut.

A follow-up dermatology appointment was organized to treat underlying fungal infection. As apparent in this case, the patient's underlying delusional beliefs acted as a smokescreen for the physicians in charge of her care. They stopped the physicians from suspecting that her leg pain was organic rather than a translated somatization or distorted perception due to her mental disorder, while they were one step away from uncovering the real cause of her reported pain. This brings out the notion of how psychiatric patients with physical complaints are at risk of being overlooked or missed when they show up to any doctor. A major factor that plays a role in this unfortunate outcome is the stigma towards such patients and the resulting attitude of the doctors treating them.

## 3. Discussion

### 3.1. A Nonneglected Body

In psychiatry, diagnostic examination is largely dominated by verbal “material”; if the body expresses itself both through its symptoms and behaviors (for instance, psychomotor retardation, bizarre remarks, rituals, possession, and hyperreligiosity), it is hardly the object of a direct investigation, mediated by a codified examination technique such as in neurology or cardiology. The examination is immediately situated as a therapeutic act, insofar as it is part of an interpersonal relationship, the modalities of which largely involve the continuation of the care, even the future of the subject's relations with the health care system or mental health team. In addition, psychiatric examination is carried out in very diverse circumstances, which influence the expression of the patient's request and the attitude of the psychiatrist.

In the context of psychiatry, the body has sometimes been taboo. The body must once again become the place of encounter and of specific care depending on the psychopathology: for example, in the indications of meditation, yoga, and sports activity, where it is far from being a technique with direct effects on the body only and proves to be a powerful organizer and at the same time physiological, transferential, and psychopathological.

In summary, psychiatry without a body does not exist! As clinicians involved in medical education in mental health, we must train psychiatrists capable of practicing psychiatry from head to toe. The clinician should take advantage of this skill which distinguishes him from psychologists. In a certain organisation, the mental health team with nurses, physiotherapist, physical trainers, pharmacist, and occupational therapist is crucial. The goal is to be the defender of the human or therapeutic relationship by giving back a place to the body which is respected, not neglected, and able to make sense of other symptoms of human suffering.

## 4. Conclusion

In today's world of super specialization and compartmentalization of medical expertise, there is a tendency to refer known psychiatric patients to their respective psychiatric specialists regardless of their presenting complaint. We can also attribute this to the innate bias in all of us as human beings. The psychiatrist then sees the patient with the mindset of managing the mental disorder, since other medical or surgical issues were supposedly taken care of. This type of bias can lead to a negative impact on the patient health.

Many scientific journals have reported an association between mental illness and poor physical health [4]. Psychiatric patients have high rates of physical illnesses, most of which go undetected. They also experience difficulties in taking care of themselves, do not eat well, are not compliant with treatments, and have side effects with psychotropic treatments [5]. These findings have led to recommendations for health professionals to ensure better medical screening and treatment of psychiatric patients. Unfortunately, there is a lot more needed to be done. The excessive morbidity and mortality among individuals with mental health problems continue unabated, with those in psychiatric outpatient care nearly twice as likely to die as the general population [6].

One way of dealing with this issue is by emphasizing the significance of performing a physical examination for all presented somatic complaints of psychiatric patients to exclude the possibility of it being a somatic issue [7, 8]. In addition, an implementation of educational methods to improve the perception of psychiatric patients by medical students at schools should be considered [9, 10]. Moreover, work is needed to enhance the awareness of nonpsychiatrist physicians to the possible consequences of prejudiced attitudes towards individuals with a mental disorder. Making physical examination part of the competency framework for specialization in psychiatry can have a considerable influence in improving patient care. Our case report illustrates that psychiatrists are clinical scientists who must be attentive to clinical signs from head to toe and better from mind to toe.

## Data Availability

The case described is a patient that is followed up in Hôpital Notre-Dame, Montreal, Quebec, Canada; all supporting documents can be found and accessed in her hospital file.
